# Comment on Chowdhury et al. Impact of Molecular Testing on Surgical Decision-Making in Indeterminate Thyroid Nodules: A Systematic Review and Meta-Analysis of Recent Advancements. *Cancers* 2025, *17*, 1156

**DOI:** 10.3390/cancers18142295

**Published:** 2026-07-16

**Authors:** Atanas Kaykov, Corey Geurink, Joshua Klopper

**Affiliations:** Veracyte, Inc., 6000 Shoreline Court, Suite 300, South San Francisco, CA 94080, USA

The manuscript by Chowdhury et al. is intended to be a systematic review and meta-analysis of the surgical avoidance rate in patients with indeterminate (Bethesda III or IV) thyroid nodules that undergo molecular testing by one of the three major commercially available testing platforms in the United States: The Afirma Gene Expression Classifier or Genomic Sequencing Classifier (GSC), ThyroSeq V2 or V3, and ThyGenX/ThyraMIR. The study concludes that ThyGenX/ThyraMIR had the highest (68.6%), while the Afirma platforms had the lowest surgical avoidance rate (50.6%) of the three platforms tested. This data significantly contrasts with a meta-analysis of thirteen independent studies, which showed that in a combined cohort of 1325 patients with Afirma GSC-Benign results (67% benign call rate), only 6% underwent surgery [1,2]. Additionally, Ahmadi et al. recently reported the analysis of a multi-institutional study for a cohort of 528 Afirma-GSC Benign patients followed up to 6 years (23 months median follow-up), showing that there was a 63% benign call rate, with only 12.6% of patients having surgery (87.4% surgical avoidance rate) [3]. In both studies, some patients with Afirma GSC-Suspicious results also avoided immediate surgery (22% and 32%, respectively), thus increasing the overall surgical avoidance rate.

Unfortunately, Chowdhury et al. suffer from significant methodological flaws and inconsistent data analysis, rendering the findings and conclusions inaccurate. Although the materials and methods section of the article states only Bethesda III and IV indeterminate nodules were to be included, data for Bethesda I, II, V, and VI nodules were analyzed in some studies (Selvaggi et al. 2021 and Sirotnikov et al. 2024) [4,5]. Moreover, despite explicitly excluding conference abstracts, four abstracts without peer-reviewed manuscripts were included (Akinsanya et al., 2022; Dantev et al., 2022; Yang et al., 2020; Bao et al., 2022). Additionally, in some studies, nodules that did not undergo molecular testing or did not have molecular testing results reported were included in the total number of samples analyzed, significantly decreasing the surgical avoidance rate for the Afirma-GSC cohort. In contrast, these nodules were appropriately excluded from other studies. Patients with nodules resected after follow-up were omitted, thereby inflating the surgical avoidance rate for ThyGenX/ThyraMIR (Tumati et al. 2024) [6]. Finally, for two studies, events of surgical avoidance were calculated by dividing patients avoiding surgery by all nodules in the cohort. As there were several patients with multiple nodules, this results in a reduced rate of surgical avoidance for Afirma-GSC.

Chowdhury et al. describe the use of Covidence, a systematic review software tool, to aid data extraction from the selected articles, and two author reviewers performing full-text reviews of potentially eligible studies. We find it difficult to understand how careful author review of the full text could have allowed the inclusion of four abstracts out of 31 eligible studies.

In summary, given the clear methodological and analytical flaws, the Chowdhury et al. study arrives at erroneous results and conclusions. Of particular concern is the potential use of such data to influence clinical decision-making for readers when choosing a molecular testing platform. While acknowledging that no study is perfect and all have limitations, Chowdhury et al. demonstrate that critical appraisal of any manuscript is necessary to ensure information relayed is as accurate and useful as possible when informing clinicians in their practice. We appreciate the opportunity to provide this commentary as a part of this important review process.

## Data Availability

No new data were created or analyzed in this study. Data sharing is not applicable to this article.
